# Multimodal Floral Signals and Moth Foraging Decisions

**DOI:** 10.1371/journal.pone.0072809

**Published:** 2013-08-21

**Authors:** Jeffrey A. Riffell, Ruben Alarcón

**Affiliations:** 1 Department of Biology, University of Washington, Seattle, Washington, United States of America; 2 Biology Program, California State University Channel Islands, Camarillo, California, United States of America; Royal Holloway University of London, United Kingdom

## Abstract

**Background:**

Combinations of floral traits – which operate as attractive signals to pollinators – act on multiple sensory modalities. For *Manduca sexta* hawkmoths, how learning modifies foraging decisions in response to those traits remains untested, and the contribution of visual and olfactory floral displays on behavior remains unclear.

**Methodology/Principal Findings:**

Using *M. sexta* and the floral traits of two important nectar resources in southwestern USA, *Datura wrightii* and *Agave palmeri*, we examined the relative importance of olfactory and visual signals. Natural visual and olfactory cues from *D. wrightii* and *A. palmeri* flowers permits testing the cues at their native intensities and composition – a contrast to many studies that have used artificial stimuli (essential oils, single odorants) that are less ecologically relevant. Results from a series of two-choice assays where the olfactory and visual floral displays were manipulated showed that naïve hawkmoths preferred flowers displaying both olfactory and visual cues. Furthermore, experiments using *A. palmeri* flowers – a species that is not very attractive to hawkmoths – showed that the visual and olfactory displays did not have synergistic effects. The combination of olfactory and visual display of *D. wrightii*, however – a flower that is highly attractive to naïve hawkmoths – did influence the time moths spent feeding from the flowers. The importance of the olfactory and visual signals were further demonstrated in learning experiments in which experienced moths, when exposed to uncoupled floral displays, ultimately chose flowers based on the previously experienced olfactory, and not visual, signals. These moths, however, had significantly longer decision times than moths exposed to coupled floral displays.

**Conclusions/Significance:**

These results highlight the importance of specific sensory modalities for foraging hawkmoths while also suggesting that they learn the floral displays as combinatorial signals and use the integrated floral traits from their memory traces to mediate future foraging decisions.

## Introduction

Multimodal signals – where the signaler uses two or more signals that operate on different modalities of the ‘receiver’ – have been shown to mediate a variety of critical ecological and evolutionary processes, including sexual selection and mate choice (reviewed by [1], [2]), predator-prey interactions [3], and plant-pollinator interactions [4]–[6]. Floral traits, including color, odor, and morphology, are excellent examples of multiple sensory “advertisements” that signal to pollinators the location of important food resources [7]. Pollination syndromes, or unique combinations of floral traits, are hypothesized to reflect adaptations of flowers for specific taxonomic classes of pollinators and are frequently used to describe floral morphology in relation to their purported agent of selection [8]–[11]. For instance, bird-pollinated flowers are predicted to have red, scentless flowers with copious amounts of nectar, and moth-pollinated flowers are predicted to be white, with long narrow corolla tubes and scented in the evening. Phylogenetic and morphological studies have demonstrated that these floral trait combinations have evolved independently within many different plant families [12]–[15]. The convergence of floral traits between distantly related floral species may suggest that the traits synergize to attract the certain pollinators [16]–[18] or, alternately, the genetic pathways for those traits are linked [19]. Nonetheless, pollinator attraction to a certain floral trait, or combination of traits, will operate as a strong selective pressure on the plant species. In particular, night-blooming plants adapted to hawkmoth pollination show a convergence of common floral features (e.g., fragrant nocturnal scent emissions, highly reflective corollas) [20]. The commonality of these floral features makes night blooming plants excellent models in which to examine plant-pollinator interactions and the behavioral effects of floral traits.

Multimodal floral signals are critical in mediating pollinator behaviors [14], [17], [18], [21], [22], with certain combinations of floral traits (odor, visual display) proving useful for predicting specific pollinator taxa and the relative importance of nocturnal and diurnal insect pollinators [12], [20], [23]. For example, the visual floral cue was found to be important in mediating feeding responses in bumblebees (*Bombus impatiens*), but learning the combination of scent and visual floral signals increased the discriminability of artificial flowers and increased the accuracy of foraging decisions [24]. In both wild and naïve *Manduca sexta* moths the simultaneous presence of olfactory and visual signals were necessary to elicit feeding behaviors [17], [18]. By contrast, for many pollinators the floral signals elicited a hierarchical behavioral response. For instance, color is more important than odor for the diurnal *Vanessa indica* butterfly and the *Macroglossum stellatarum* hawkmoth when feeding from artificial flowers [25], [26], while for plasterer bees (*Colletes cunicularius*) the floral odor is more important than color [27]. A given floral trait can thus have a dominant effect on behavior, or have an additive or synergistic effect with other floral traits, depending upon the importance of the pollinator sensory modalities mediating the flower visitations.

Our understanding of the effects of multimodal stimuli on pollinator behavior has benefited from studies using highly attractive stimuli and more recent studies on the effects of learning in modulating behavioral responses. In particular, research using hawkmoths has elegantly demonstrated the interaction between olfactory, visual, and somatosensory signals in mediating flower attraction and handling [16]–[18], [21], [25], [28]–[32]. In a seminal series of experiments, Raguso and Willis demonstrated the interaction between olfactory and visual cues in a laboratory setting [17], and further demonstrated these interactions in the field [18]. These studies, using either paper or bagged flowers as the visual stimuli, and scent from attractive flowers (*Oenothera neomexicana* and *Datura wrightii*), showed that the visual stimulus was critical for initiating the proboscis extension response in *Manduca* sp. [17], [18]. Further studies in the laboratory have also demonstrated the synergy between olfactory and visual cues in mediating moth feeding behaviors [28], [32] and the importance of temporal and spatial contiguity of the stimuli [28]. In addition, experiments by Goyret and coworkers have elegantly teased apart the relative importance of visual and sematosensory stimuli in mediating feeding responses in *M. sexta* moths [16], [29]. Multimodal displays have also been shown to influence a pollinator's learning ability. For example, using artificial flowers, *M. sexta* moths were shown to learn the association between a scented visual stimulus and a sugar reward [33], as well as a color stimulus and sugar reward [34]. This research relates to work with bumblebees, where bees were shown to learn more quickly in response to a multimodal than single modal displays (eg, scent and color versus scent or color alone), and that display complexity increased foraging efficiency [24], [35]. Taken together, these studies demonstrate that sensory stimuli synergize with one another to drive behavior (but see [34]), and that learning can modulate the responses to cues that are initially unattractive. However, there are a number of gaps in our understanding of how combinations of floral traits influence *M. sexta* foraging decisions. For instance, studies have often used olfactory stimuli that do not realistically simulate the natural volatile emissions from flowers, or have used visual stimuli from paper flowers that do not reflect the morphological and visual complexity of flowers. Although artificial stimuli provide meaningful control of visual and olfactory displays, thereby providing a first principles approach for understanding how these modalities interact, it remains unclear whether behaviors elicited by natural floral stimuli are similar to artificial stimuli. In addition, the interactions of individual traits and trait combinations from attractive flowers compared to less attractive flowers in influencing moth feeding responses has yet to be fully explored. Last, how learning modifies these interactions also remains unclear.

**Figure 1:**
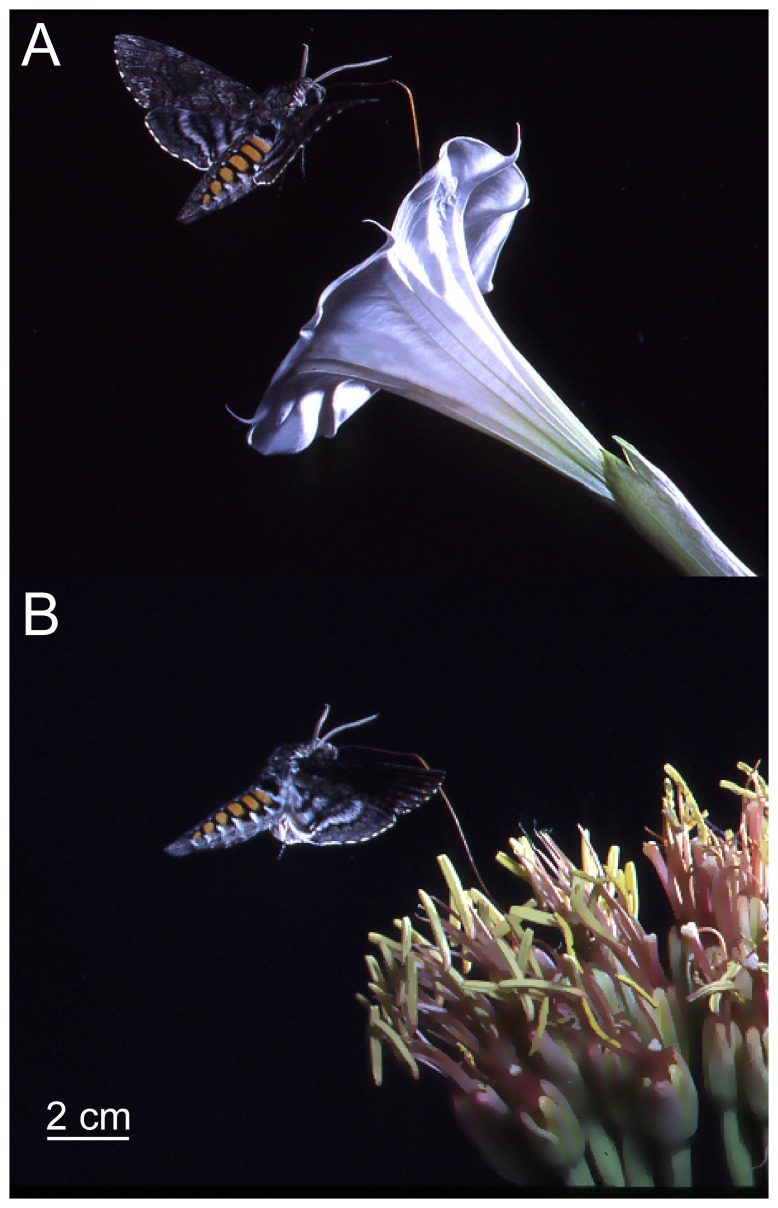
In southern AZ, *M. sexta* moths use the nectar resources of two plants: *A. palmeri* and *D. wrightii*. (**A**) The *D. wrightii* flower exhibits classic characteristics of moth-adaptation with reflective corolla, sweet-smelling perfume, and sucrose-dominant nectar. These characteristics elicit an innate feeding behavior in *M. sexta*. (**B**) *M. sexta* also nectars from *A. palmeri* flowers. Moths learn to utilize *A. palmeri* through association of the floral scent and nectar.

In the current study, we present different semi-natural visual and olfactory signals to the *M. sexta* hawkmoth using flowers from *Datura wrightii* (Solanaceae) and *Agave palmeri* (Agavaceae) (Fig. 1). *D. wrightii* possesses the typical phenotype of hawkmoth-adapted flowers, including nocturnal anthesis, intense and sweet fragrance, and reflective coloration; whereas *A. palmeri* exhibits the phenotype of bat-adapted flowers with its abundant hexose-rich nectar, robust morphology and strong pungent odor. *M. sexta* hawkmoths visit *D. wrightii* flowers based on an instinctive bias, but in the Sonoran Desert of southern Arizona they also learn to feed from *A. palmeri* flowers based on an association with its floral odor and nectar reward [36], [37]. While these studies were suggestive of the importance of the floral scent, the effects of the floral visual display were never explicitly tested, and the relative importance of the olfactory and visual floral display, or unique combinations of the two displays, remains unknown. Here in this study, we conduct similar experimental manipulations performed by other investigators [17], [18], [28], [37] but using the natural visual and olfactory displays of *D.wrightii* (highly attractive) and *A. palmeri* (less attractive) flowers to examine the contribution of olfactory and visual signals in moth foraging behaviors. In addition, we examine how the two modalities interact in experienced animals.

## Materials and Methods

### Study system

*M. sexta* is a large hawkmoth (10 cm body length) with a widespread distribution, from South America to North America. In the southwestern USA *M. sexta* oviposits on *Datura* spp. and (more rarely) *Proboscidea* spp. (Martyniaceae) [38]. *D. wrightii* however, not only operates as the hostplant for *M. sexta* but also an important nectar resource for adults. During the monsoon season (July-September) in southern Arizona *D. wrightii* produce low numbers of large (10 cm diameter), white, tubular flowers each night. Flowers are highly reflective in the 400 to 800 nm range of the light spectrum, and only open for a single night secreting concentrated nectar (ca. 60 µl; ca. 25% sugar content) [37], [39], [40]. *M. sexta* is the primary pollinator of this plant in these habitats (Fig. 1A) [36], [41]. When *D. wrightii* is not abundant, however, *M. sexta* adults feed from flowers of the *A. palmeri*. *A. palmeri* exhibits floral traits typical of bat pollination, with hexose-rich nectar, aliphatic ester-dominated volatiles that produce a rotten fruit odor. Moreover, *A. palmeri* has a lower flower reflectance than *D. wrightii* in the 450 to 600 nm range of the visible spectrum (Fig. 1B). *A. palmeri* flowers form dense clusters, or umbels, typically 1–5 per branch, that occur at 2–5 m height above the ground. These flowers produce copious amounts of nectar (ca. 200–300 µl per flower each night) that *M. sexta* moths learn to associate with the rotten fruit odor of the flowers [37]. These two flower species, one eliciting an innate behavior and the other a learned, thereby provide an excellent system in which to determine the contribution of olfactory and visual signals in mediating feeding behaviors in naïve and experienced moths.

### Characterization and manipulation of flower odor and visual traits

#### Flower reflectance and bagging

**Figure 2:**
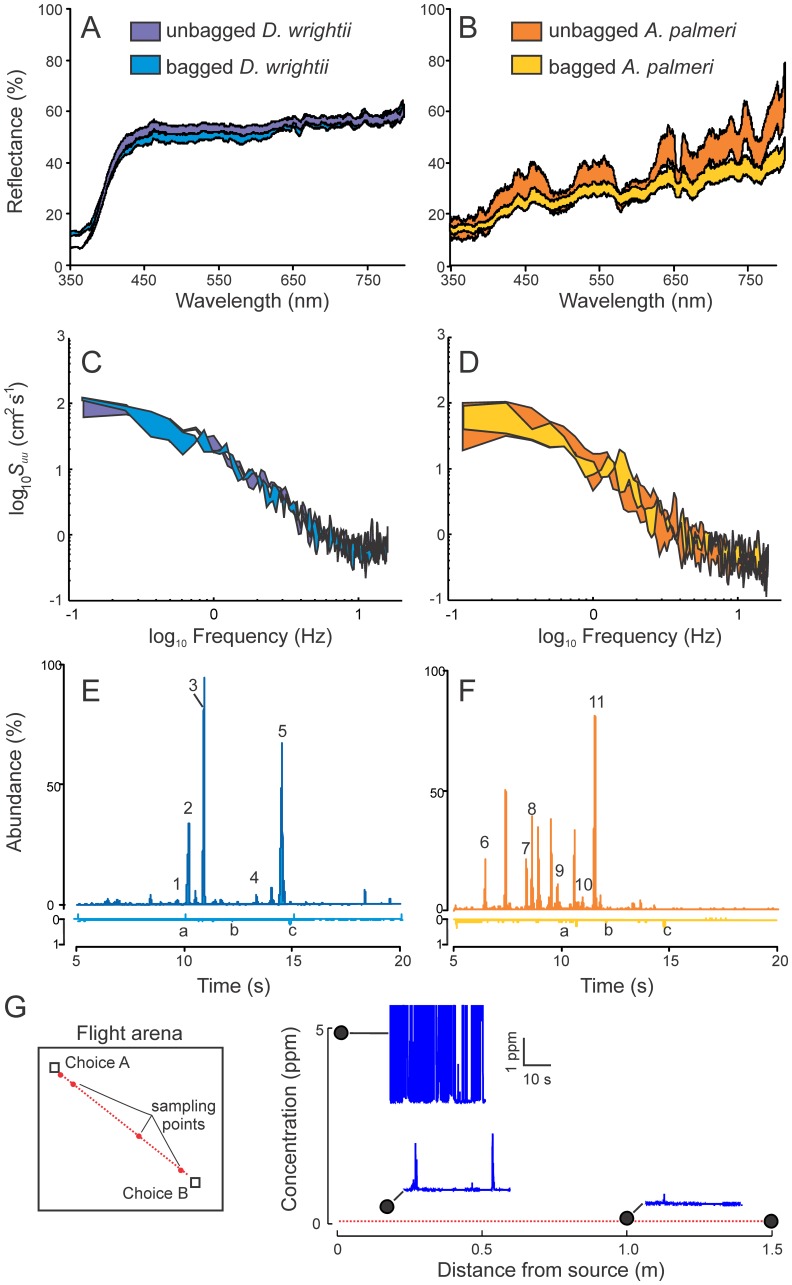
Characterization of the effects of bagging flowers on floral scent emissions, reflectance and gross morphology. (**A**) *D. wrightii* corolla reflectances of unbagged (dark blue) and bagged (light blue) flowers. (**B**) *A. palmeri* petal reflectance of unbagged (orange) and bagged (yellow) flowers. For both flower species, bagging had little effect on floral reflectance. (**C**) The power spectrum of wind velocity fluctuations produced from a bagged (light blue) and unbagged (dark blue) *D. wrightii* flowers in a wind tunnel. (**D**) The power spectrum of wind velocity fluctuations produced from bagged (yellow) and unbagged (orange) *A. palmeri* umbels. For both *D. wrightii* and *A. palmeri* flowers, bagging had no effect on the turbulent wind fluctuations or energy cascade. (**E**) GCMS ion chromatograms of the captured headspace volatiles emitted from unbagged (dark blue) and bagged (light blue) *D. wrightii* flowers. Major constituents of *D. wrightii* floral headspace scent shown in the total ion chromatogram are monoterpenoids *β*-myrcene (1), *E-β*-ocimene (3), and geraniol (5), aromatics including benzyl alcohol (2) and methyl salicylate (4). Letters denote contamination from the polyacetate bag. (**F**) Ion chromatograms of the headspace volatiles from bagged (yellow) and unbagged (orange) *A. palmeri* umbels. Major constituents of *A. palmeri* floral odor shown in the total ion chromatogram are monoterpenoids *α*-pinene (7), camphene (8), and esters such as ethyl isovalerate and analogs (6, 9) and ethyl sorbate isomers (10 and 11). Letters denote contamination from the polyacetate bag. (**G**) Flower volatile concentrations measured at specific locations in a flight arena from the emitting flower using a mini photoionization detector. (*Left*) Schematic of the flight arena and sampling locations from the flower. (*Right*) Mean volatile concentrations at the locations from the flower. Volatile concentrations drop rapidly with increasing distance from the flower until at 1 m from the flower the concentration are <0.05% of the intensity near the source. Insets are photoionization traces through time, at sample points 0.05, 0.2, and 1.0 m from the source. Dashed line indicates the volatile background concentration.

Description of the different experimental treatments can be found in Table 1. To provide components of the flower visual display (reflectance and gross morphology) while permitting manipulation of the flower odors and preventing nectar access, flowers were enclosed in transparent polyacetate bags (Reynolds, Inc., Richmond VA, USA) (sensu [18]). To determine if the bag modified the flower reflectance and shape, two series of measurements were conducted: the first examining the flower spectral properties, and the second by examining the effects of the flower shape on the laminar wind flow in a wind tunnel. First, floral spectral reflectance in the range 300–700 nm was determined for ten flowers per plant, with an Ocean Optics USB 4000 UV-VIS spectrometer (Ocean Optics Inc., Dunedin, Fla.) and fiber optic reflection probe (400 µm) held at 45° angle to the petal surface. The light source used was an Ocean Optics DT-MINI-2-GS light source with a spectral range of ca. 200–1100 nm attached to a PC running OODBase32 software (Ocean Optics Inc., Dunedin, FL, USA). We used an Ocean Optics WS-1 diffuse reflectance standard to calibrate the spectrometer. Flowers were placed over a black background, and the integration sphere, fitted with a 1.5 cm diameter sampling port, was placed over the adaxial, distal portion of the flower corolla to capture the transmitted light scattered by its textured surface. Data were collected from flowers covered with, and without, the bag and expressed as percent reflectance relative to the white reflectance standard. This standard reflected evenly in all non-UV wavelengths, with a 2% drop-off from 300 to 350 nm. The reflectance of the black background was negligible (0.5–1% of standard) for all wavelengths tested and thus did not contribute any artifacts to the spectral measurements. Results of these determinations revealed that the polyacetate bags decreased the flower brightness by ca. 5–10% (Fig. 2A,B) but did not modify the shape of the spectral reflectance curves.

**Table 1:** Two-choice experimental treatments.

Treatment:	Expt. #	Moths tested:	Flower A	Flower B
***Naïve moths - single modality tests***				
No cue	1.	20	Shade-cloth covered paper flower	Shade-cloth covered paper flower
				
Odor display	2.	20	Shade-cloth covered *D. wrightii*	Shade-cloth covered paper flower
				
Visual display	3.	20	Paper flower	Shade-cloth covered paper flower
				
Odor *vs.* Visual	4.	20	Shade-cloth covered *D. wrightii*	Paper flower
***Naïve moths - single and multiple cues***				
Odor display				
	5.	23	Paper flower + *A. palmeri* odor	Paper flower + *D. wrightii* odor
	6.	30	Paper flower (control)	Paper flower + *D. wrightii* odor
	7.	21	Paper flower (control)	Paper flower + *A. palmeri* odor
Visual display				
	8.	16	Paper flower (control)	Paper flower (control)
	9.	33	*A. palmeri* visual	*D. wrightii* visual
	10.	20	Paper flower (control)	*D. wrightii* visual
	11.	20	Paper flower (control)	*A. palmeri* visual
Visual *vs.* Odor				
	12.	16	*D. wrightii* visual	Paper flower + *D. wrightii* odor
	13.	20	*A. palmeri* visual	Paper flower + *A. palmeri* odor
Odor *vs.* Visual+Odor				
	14.	20	Paper flower + *D. wrightii* odor	*D. wrightii* visual + *D. wrightii* odor
	15.	20	Paper flower + *A. palmeri* odor	*A. palmeri* visual + *A. palmeri* odor
Visual *vs.* Visual+Odor				
	16.	20	*D. wrightii* visual	*D. wrightii* visual + *D. wrightii* odor
	17.	20	*A. palmeri* visual	*A. palmeri* visual + *A. palmeri* odor
***Naïve moths - uniform odor***				
	18.	20	*A. palmeri* visual + *A. palmeri* odor	*D. wrightii* visual + *A. palmeri* odor
	19.	20	*A. palmeri* visual + *D. wrightii* odor	*D. wrightii* visual + *D. wrightii* odor
***Experienced moths - coupled floral cues***				
*D. wrightii*-experienced*	20.	20	*D. wrightii* visual + *D. wrightii* odor	*A. palmeri* visual + *A. palmeri* odor
*A. palmeri*-experienced*	21.	20	*D. wrightii* visual + *D. wrightii* odor	*A. palmeri* visual + *A. palmeri* odor
***Experienced moths - uncoupled floral cues***				
*D. wrightii*-experienced*	22.	24	*D. wrightii* visual + *A. palmeri* odor	*A. palmeri* visual + *D. wrightii* odor
*A. palmeri*-experienced*	23.	24	*D. wrightii* visual + *A. palmeri* odor	*A. palmeri* visual + *D. wrightii* odor
***Naïve moths -coupled floral cues***				
Naïve moths (cage control)	24.	24	*D. wrightii* visual + *D. wrightii* odor	*A. palmeri* visual + *A. palmeri* odor

* experienced moths were those that had encountered both the floral nectar and floral visual and olfactory traits.

In the second series of determinations, the effects of the bags on flower shape were examined in a laboratory wind tunnel (Plexiglas, L×W×H = 4.0×1.5×1.5 m). These measurements allowed determination of how the bag modified the airflow around the flower, which may in turn affect the floral odor plume. Air was forced into the upwind end of the tunnel through a carbon filter and exhausted at the downwind end through a duct vented into a laboratory fume hood. Flowers, in the upwind portion of the tunnel, were held in the center of the wind tunnel by a thin (ca. 2 mm diameter) metal rod and clamp to avoid turbulent production. A 3D sonic anemometer (81000, RM Young Co., Traverse City, MI, USA), sampling at 32 Hz, was placed 2 m downwind from the flower. As measured by the anemometer, the wind speed was 15 cm/s and turbulent shear stresses of ca. 0.001 N/m^2^ occurred along the principal axes (*u, w*). For both *A. palmeri* and *D. wrightii* flowers, bagging had no significant effect on the turbulent Reynolds stresses and inertial eddy cascades produced by the flowers (Fig. 2C,D; paired *t-*test for both species: *t*_11_≤−0.45, *P*≥0.88) thereby demonstrating that bagging did not grossly modify flower shape.

#### Flower odor quantification and manipulation

Bagging of the flowers further provided the means by which to manipulate the odor around the flowers. For example, the *A. palmeri* floral scent could be presented with the *D. wrightii* visual stimulus, and *vice versa*, thereby allowing decoupling between the floral visual and olfactory traits. To quantify the scent emissions from unbagged flowers, and to determine the extent to which the floral odor escaped the bag, headspace collections were conducted (sensu [37], [40]). Briefly, bagged and unbagged flowers (*N = *6–10 scent collections for each bag treatment and flower species) were enclosed in oven bags (Reynolds®, Reynolds Kitchens. Richmond, VA, USA) cinched at 500 mL volumes with plastic ties. Portable diaphragm pumps (10D1125, Gast Manufacturing Inc., Benton Harbor, MI, USA) were used to pull fragrant headspace air through borosilicate glass tubes packed with scent traps (100 mg of Super Q; mesh size 80–100, Alltech, Deerfield, IL, USA) at a flow rate of 500 mL/min. Scent collections began in the evenings (sunset) and continued overnight for 10 h. Trapped volatiles were eluted from sorbent cartridges using 600 µL of HPLC-grade hexane. Each sample was stored in a 2 mL borosilicate glass vial with a Teflon-lined cap at –80 °C until analysis. Volatile sample (1 µL) was analyzed using gas chromatography with flame-ionization detection (GC-FID) and gas chromatography-time-of-flight mass spectrometry (GC-ToF-MS). The GC-ToF-MS system consisted of an HP 6890 (Agilent Technologies, Palo Alto, CA, USA) GC and a Waters TOF-MS (Waters-Micromass, Millford, MA, USA). A DB1 GC column (30 m, 0.25 mm, 0.25 µm; J&W Scientific, Folsom, CA, USA) was used, and helium was used as carrier gas at constant flow of 1 ml/min. The initial oven temperature was 50° C for 5 min, followed by a heating gradient of 6° C/min to 230° C, which was held isothermally for 6 min. Peaks were identified using ToF-MS with 70 eV electron-impact ionization. Chromatogram peaks were identified tentatively with the aid of the NIST mass spectral library (ca. 120,000 spectra) and verified by chromatography with authentic standards or known components of essential oils. Floral emission rates were quantified using a GC-FID system consisting of a Shimadzu model 14A GC (Columbia, MD USA) equipped with a flame ionization detector. As with the GC-ToF-MS, a DB1 column and similar temperature parameters were used. Peak area for each odorant was quantified using an external five-point standard (0.01 ng to 100 ng) of synthetic odorants and expressed in units of ng per flower per h.

In addition to quantification of the emissions from bagged and unbagged flowers, we quantified the floral emissions from single freshly cut flowers enclosed in 3 L glass jars placed outside of the flight arenas used in behavioral tests (see below). Charcoal-filtered air was pumped into the jar at 0.02 L/min and conducted from the jar through 2 m of Teflon tubing (2 mm I.D.) to either a paper flower, or a bagged flower, in the flight arena. As in the headspace collections for the bagged and unbagged flowers, the emissions were collected for 10 h by the dynamic sorption method and subsequently analyzed via GC-FID and GC-ToF-MS. Similar to our previous results [37], [42], floral scent at this flow rate produced emissions from artificial flowers similar to those from natural flowers (58.65 ± 10.4 ng/h and 72.8 ± 13.8 ng/h for artificial and real flowers, respectively; unpaired *t*-test: *t*_15_ = −14.1, *P* = 0.43). By contrast, bagging of the flowers to prevent scent emissions caused a significant 500-fold reduction in scent emissions (0.12 ± 0.03 ng/h; unpaired *t-*test: *t*_15_>−58.5, *P*<0.001: Fig. 2E,F). The paper flowers thus provided moths an attractive visual display but lacked the morphological and tactile characteristics that are attractive to foraging hawkmoths [16], [28].

To determine the spatial distribution of the scent from one of the flower “choices” in the arena, a mini photoionization detector (miniPID, Aurora Scientific Inc., Aurora, Ontario, Canada) was used to quantify the floral volatiles. Concentrations from the flower plume were measured at distances 0.05, 0.2, 1.0, and 1.5 m from the source, at the same height above the substratum as the flower. Measurements were sampled at 300 Hz for 30 s and digitally recorded using Matlab software (Mathworks Inc., Natick, MA USA). Based on these measurements, volatile concentrations decay rapidly until reaching near-background levels 1.5 m away from the source (Fig. 2G). Together, these floral manipulations allowed explicit testing of semi-natural single traits or trait combinations in mediating foraging decisions in *M. sexta* hawkmoths.

### Behavioral two-choice experiments – naïve animals

#### Experimental setting

A series of experiments were conducted with naïve laboratory-reared male moths to establish the relative contribution between different combinations of sensory modalities in mediating flower-feeding behavior (see Table 1 for treatment details). As in Riffell *et al.* (2008), male moths that had eclosed 3 days prior to testing were used and moths were only used once. Moths were exposed to ambient light conditions during the summer months when *A. palmeri* and *D. wrightii* are flowering, and maintained at 75–85% RH. Behavioral testing of the moths began once they had entered scotophase (19:30 PST) and continued for up to 3 h. Experiments were conducted by releasing single moths into a flight arena (1.8×1.8×1.8 m) containing two different flower treatments. The flower treatments were positioned at a height of 1 m from the ground, and were randomly placed in the arena and spaced 1.5 m apart. Measured response variables were the treatment at which the first proboscis extension into the flower corolla took place, and the attempted feeding time. Here, we describe feeding time as the total time that the moth had its proboscis in the flower corolla. Each trial was 10 minutes in duration or lasted until the moth stopped flying for more than 3 min. The moth was then removed from the cage, and after an interval of at least 5 min, another moth was released. A total of 511 moths were used in these experiments.

**Figure 3:**
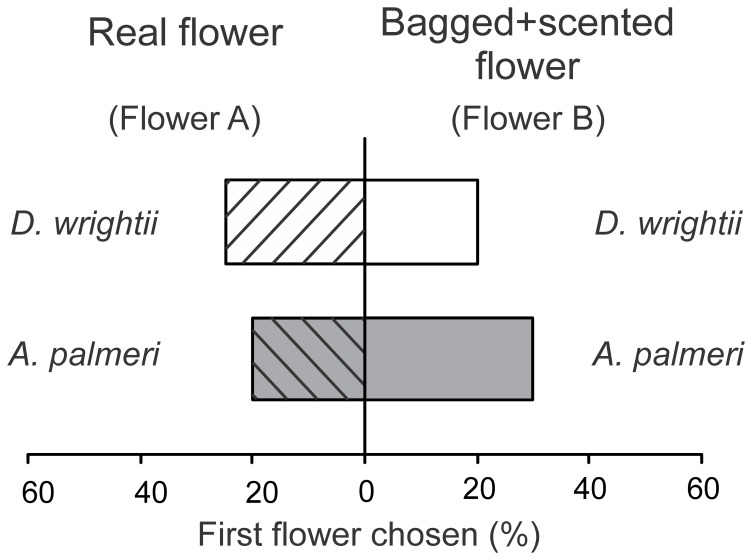
Two-choice experiments examining the visual and olfactory floral preferences of naïve male *M. sexta* moths in response to live flowers or scented bagged flowers. (*Top*) The percentage of moths that chose the live *D. wright*ii flower (Flower A) or a bagged *D. wrightii* flower with *D. wrightii* scent (Flower B). (*Bottom*) The percentage of moths that chose the live *A. palmeri* umbel (Flower A) or a bagged umbel with scent (Flower B). 20 moths were used in each two-choice experiment. In both experimental series, there were no significant differences in the first flower chosen (G-test: *P*>0.71). *D. wrightii* flower cues (odor and visual) are represented by white bars, *A. palmeri* flower cues (odor and visual) are represented by grey bars, and hashed bars represent the real flowers.

The individual flower treatments used in the two-choice experiments were manipulated to provide either single or multimodal sensory signals to the moth, while also permitting the ability to exchange the two flower species' visual and olfactory traits. Using naïve moths, the manipulated two-choice treatments are similar to those used in previous studies [17], [37], but use of the *D. wrightii* (highly attractive) and *A. palmeri* (less attractive) flowers provide naturalistic stimuli to test the effects of floral traits on moth behavior while allowing comparison of responses to both floral species. When using real flowers, a single freshly cut *D. wrightii* flower and an *A. palmeri* umbel (7–12 flowers) were used in each experiment. *D. wrightii* flowers and *A. palmeri* umbels used in the experiments were approximately the same size (8–15 cm) and have similar scent emissions [37]. We first examined the moth's response to single modality cues by covering flowers with a green shade cloth (KG6; DeWitt Co., Sikeston, MO USA), in a series of two-choice experiments. This manipulation prevented the moths from seeing a high contrast object, while also allowing testing of the flower odors. Moths were exposed to treatments including flowers covered with the shade cloth, thereby presenting the moths with only the scent, or a white, conical, paper flower – consisting of white paper cones with an opening 8 cm in diameter and a 10 cm length – that allowed testing the moth responses to a high contrast visual object. In addition, manipulation of the flower cues also allowed the testing of multimodal stimuli in two-choice experiments. To accomplish this, individual treatments used in the experiments were: (*i*) bagged flowers; (*ii*) bagged flowers with the floral odor pumped to the location of the flower corolla (see above for details); (*iii*) paper flowers; or (*iv*) paper flowers with the floral odor pumped to the location of the flower corolla. The first treatment group provided the visual display of the flower, the second treatment group provided visual and olfactory floral signals, the third treatment group provided a visual display without morphological features that might be attractive to the moths [28], [37], and the fourth treatment group provided a visual display with an olfactory signal. Paper flowers were used because moth feeding behaviors are reliant upon a visual stimulus [17], [18]. In addition, two-choice pilot experiments were conducted with 3 day-old naïve male moths to determine whether bagging of the flower and pumping the floral odor elicited different behavioral responses in comparison to unbagged flowers. Results from these pilot tests demonstrated that, for naïve moths, bagging of the flowers and pumping of the odor elicited the same level of behavior as the unbagged flowers (Fig.3; *G*-test: *G* = 0.14, *P* = 0.71).

#### Naïve moths – single and multimodality tests

**Figure 4:**
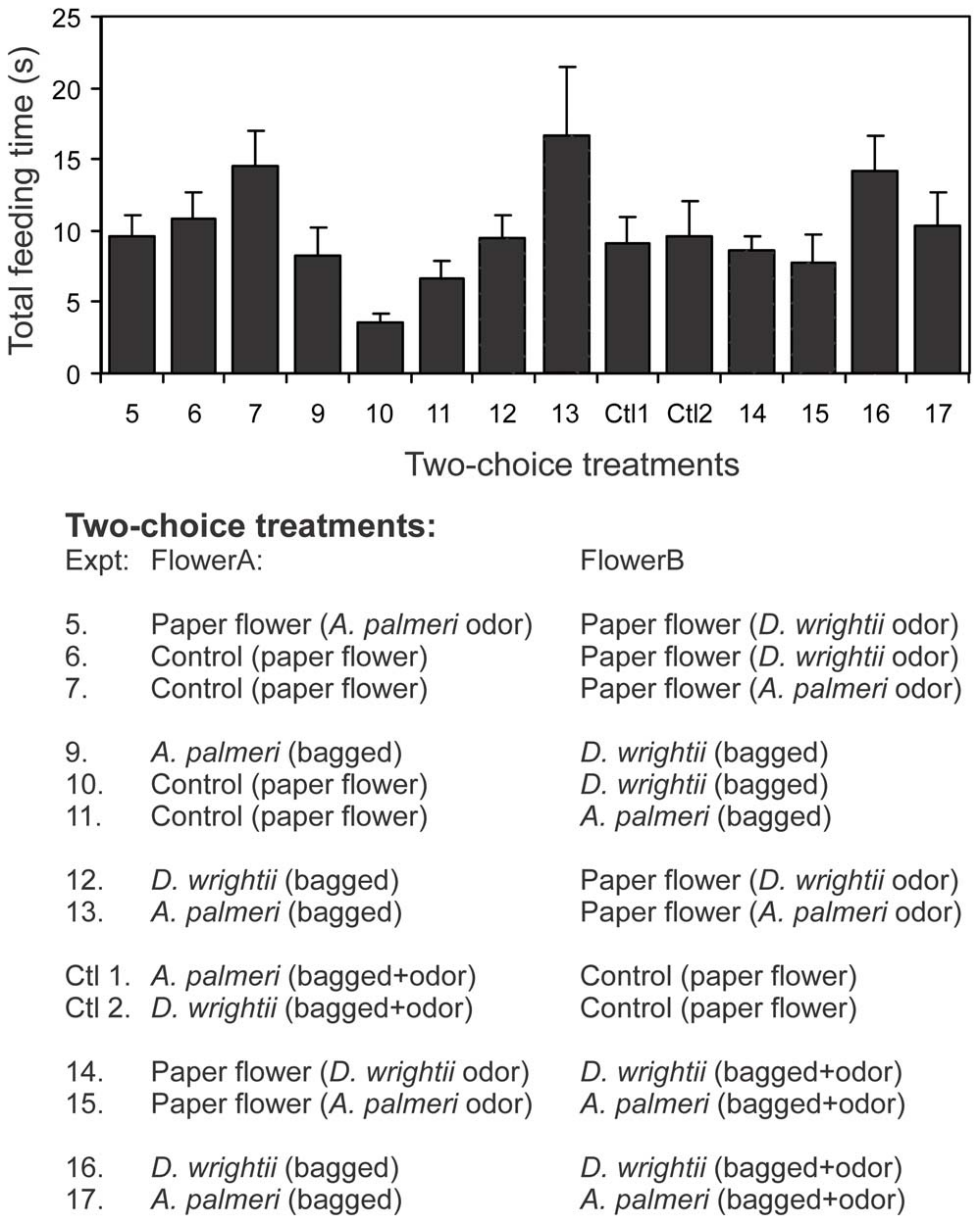
Total time *M. sexta* moths attempted to feed from flowers in two-choice experiments. There were no significant differences between mean flower feeding times between tests (one-way ANOVA: *F*_13,121_ = 0.88, *P = *0.57) or between two-choice treatment groups (post-hoc Scheffé test all comparisons: P>0.97). Note: only manipulative two-choice test values are shown for clarity. Experiments are numbered according to two-choice treatments shown in Table 1.

To examine the individual effects of natural olfactory and visual signals on moth behavior, a series of two-choice experiments were conducted: (1) A shade-cloth covered paper flower (no odor, no visual) *vs.* a shade-cloth covered paper flower (no odor, no visual); (2) A shade-cloth covered *D. wrightii* flower (odor, no visual) *vs*. a shade-clothe covered paper flower (no odor, no visual); (3) A paper flower (no odor, visual) *vs.* a shade-cloth covered paper flower (no odor, no visual); and (4) A shade-cloth covered *D. wrightii* flower (odor, no visual) *vs*. a paper flower (no visual). The presence of an odor stimulus in the same area as the visual stimulus may modify visual search behaviors (eg, olfactory “priming”; [43]). However, comparison of the feeding times between treatments with only visual stimuli versus those that have both olfactory and visual stimuli showed similar levels of responses (Fig. 4) indicating the presence of the olfactory stimulus did not substantially modify the behavioral results. These experiments allowed the testing of the relative effects of visual and olfactory cues on moth foraging decisions.

To test the effects of multimodal cues and flower species on moth foraging, another panel of two-choice experiments were conducted: (5) *A. palmeri* scented paper flower *vs*. *D. wrightii* scented paper flower; (6) paper flower (no odor) control *vs*. *D. wrightii* scented paper flower; (7) paper flower (no odor) control *vs*. *A. palmeri* scented paper flower; (8) paper flower (no odor) control *vs*. paper flower (no odor) control; (9) bagged *A. palmeri* flower (no odor) *vs*. bagged *D. wrightii* flower (no odor); (10) paper flower (no odor) control *vs*. bagged *D. wrightii* flower (no odor); or (11) paper flower (no odor) control *vs*. bagged *A. palmeri* flower (no odor) (Table 1). Experiment 5 examined the olfactory preference of the moths between flower odors, and Experiments 6 and 7 tested whether the moths would attempt to feed from a scent-emitting paper flower versus a paper flower with no scent (Table 1). Experiments 8–10 examined the visual preference of the moths between flowers (Experiment 9) and determined whether the moths would attempt to feed from the visual stimulus of a flower relative to the artificial paper flower (Experiments 10 and 11). Experiment 8 examined the importance of a visual stimulus that lacked attractive morphological features and whether odor contamination might occur (Table 1). When using freshly cut flowers, flowers were replaced after every four trials (owing to a limitation in the number of available *A. palmeri* umbels). Sixteen to thirty moths were used for each two-choice treatment.

#### Naïve moths – Multimodality tests

Two-choice experiments were conducted to examine the relative contribution of multimodal floral signals on moth feeding behaviors. Moths were exposed to one of six floral treatments (numbered consecutively from the prior experiments): (12) bagged *D. wrightii* flower (no odor) vs. *D. wrightii*-scented paper flower; (13) bagged *A. palmeri* umbel (no odor) vs. *A. palmeri*-scented paper flower; (14) *D. wrightii*-scented paper flower vs. *D. wrightii* flower (odor+visual); (15) *A. palmeri*-scented paper flower vs. *A. palmeri* umbel (odor+visual); (16) bagged *D. wrightii* flower (no odor) vs. *D. wrightii* flower (odor+visual); or (17) bagged *A. palmeri* umbel (no odor) vs. *A. palmeri* umbel (odor+visual). Experiments 12 and 13 examined the relative effects between floral odor with a paper flower visual display and the visual display of the flowers; experiments 14 and 15 examined the effects of odor+visual floral signals *versus* odor with the paper flower display; and experiments 16 and 17 examined the effects of odor+visual floral signals *versus* visual signals alone (Table 1).

To examine if a uniform odor signal in combination with two different visual signals modified naïve moth feeding preferences, two additional tests were performed: (18) *A. palmeri* floral odor was pumped to both a bagged *D. wrightii* flower and a bagged *A. palmeri* umbel; and (19) *D. wrightii* floral odor was pumped to both a bagged *D. wrightii* flower and a bagged *A. palmeri* umbel. These experiments are analogous to experiments 14 and 15 while accounting for any behavioral differences elicited by the *D. wrightii* (highly attractive) and *A. palmeri* floral odors (Table 1). Twenty to thirty moths were used for each two-choice treatment. Taken together, these experiments with naïve moths allowed the testing of the following hypotheses:

- **H_1_**: The floral visual display is necessary for moth feeding responses.
- **H_2_**: The simultaneous presence of an olfactory and visual stimulus in the flight arena increases moth behavioral responses in comparison to moths in the flight arena in the presence of stimuli activating one modality.
- **H_3_**: The olfactory and visual displays of *D. wrightii* flowers, either in isolated or together, are more attractive than *A. palmeri* displays.
- **H_4_**: *D. wrightii* and *A. palmeri* visual displays are more attractive than the paper flower display.

### Behavioral two-choice experiments – experienced animals and floral cue coupling

Experiments were conducted to evaluate the effects of learning on *M. sexta* floral preference and discrimination. Here we use the term “experience” to denote that the moths had prior contact with the flowers and the opportunity to learn to associate the flower traits with the nectar reward. In the procedure detailed above, the moths are trained analogous to an absolute conditioning procedure [44]–[46]. Absolute conditioning implies the learning of the association between a flower stimulus and its nectar reward. By contrast, learning protocols like differential conditioning – where the pollinator is exposed simultaneously to one flower with a reward, and a different flower without a reward – implies the learning of both the rewarding and non-rewarding stimuli. It is believed that different levels of attention may underlie these two different conditioning procedures, with absolute conditioning requiring less information to learn to complete the task [45]. Thus, absolute conditioning may affect the moth's ability to discriminate between perceptually closer stimuli such as two flowers that have similar traits. Because *D. wrightii* and *A. palmeri* are dissimilar in their traits [37], this learning procedure provides a first principles approach towards examining the effects of coupled and uncoupled floral stimuli on moth foraging behavior.

Moths were trained to associate the floral traits with the nectar reward by having two day old moths, 24 h prior to testing, transferred to a partially covered Plexiglas® cage (1 m^3^) and subjected to one of three treatments: (*i*) moths were exposed to an array of four cut *D. wrightii* flowers; (*ii*) moths were exposed to an *A. palmeri* umbel (7–12 flowers); or (*iii*) a group of naïve moths that were not exposed to any flowers or plant-related odors. Experiments with naïve moths that had no prior exposure to the *D. wrightii* or *A. palmeri* flowers allowed comparisons with the other treatment groups to examine the effects of flower conditioning, while also controlling for cage effects. On a given experimental evening, four to eight moths were assigned to each treatment group. Once placed in the cage, moths were observed for 0.5–1.5 h at anthesis to determine if moths fed from the flowers. Within this period, approximately 20–60% of the moths from the different treatment groups were observed to have collected nectar from the flowers.

Six hours prior to testing (while still in photophase), moths were removed from the cages containing the flowers and placed into fiberglass-screen cages (31×31×32 cm) separated according to treatment group. Behavioral testing of the experienced moths began in the evening once moths had entered scotophase (19:30 PST) and continued for up to 3 hours. Experienced moths were exposed to either treatments where the floral signals were maintained coupled (e.g., *D. wrightii* visual display with *D. wrightii* odor) or uncoupled (e.g., *D. wrightii* visual display with *A. palmeri* odor). The resulting treatment combinations were: (20) *D. wrightii*-experienced moths exposed to *D. wrightii* visual + *D. wrightii* odor vs. *A. palmeri* visual + *A. palmeri* odor; (21) *A. palmeri*-experienced moths exposed to *D. wrightii* visual + *D. wrightii* odor vs. *A. palmeri* visual + *A. palmeri* odor; (22) *D. wrightii*-experienced moths exposed to *D. wrightii* visual + *A. palmeri* odor vs. *A. palmeri* visual + *D. wrightii* odor; (23) *A. palmeri*-experienced moths exposed to *D. wrightii* visual + *A. palmeri* odor vs. *A. palmeri* visual + *D. wrightii* odor; or (24) to control for cage effects, naïve moths, placed in a cage for 24 h, are exposed to *D. wrightii* visual + *D. wrightii* odor vs. *A. palmeri* visual + *A. palmeri* odor (Table 1). Floral preference was determined based on the same criteria used for experiments with naïve moths, but in addition, the time it took the moths to begin feeding from the flowers as well as video motion analysis of flight behaviors was determined. Video images of flight tracks were captured by an overhead CCD camera (1034 by 779 pixels; 31 fps; Scout A1000-30 g; Basler Inc., Exton, PA USA) with a macro-lens (2×2 m area). The video was digitized and analyzed with a video-acquisition and motion-analysis system (Peak Motus 3D v7.2, Vicon, Los Angeles, CA, USA) and the resulting 2-D flight tracks were analyzed. Twenty to twenty-four flight tracks (from equivalent number of moths) were used for each two-choice treatment. These behavioral experiments allowed us to test the following hypothesis:

- **H_5_**: Moths respond equally to the olfactory and visual stimuli from a previously experienced flower.

### Statistical analyses

For the two-choice behavioral experiments, differences in the first flower chosen between the two individual treatments were determined with *G*-tests on a treatment group-by-treatment group basis. Based on the time spent attempting to feed from the flowers, a Response Index was calculated by (Time_FlowerB_ – Time_FlowerA_)/(Total Time_FlowerB+A_). Differences in the Response Indices between two-choice treatments were determined using unpaired *t*-tests with a Bonferroni correction to reduce type I errors. Results are presented as the percentage of moths, from the total number bioassayed, that attempted to feed from flowers. Note that, on average, only 50–70% of the tested moths will feed from a live flower [37]. Videoimages and the flight track data were used to determine the “correct decision time” – defined as the time it takes a moth to feed from the previously experienced flower – when exposed to the coupled and uncoupled flower treatments. The flight tracks were also analyzed according to the total distance of the moth flight path (sensu [47]).

## Results

As a first step in examining the contribution of olfactory and visual cues in mediating feeding responses, we tested the responses of naïve moths to floral treatments that activate only a single modality. Next, individual moths were tested in a suite of assays examining the relative importance of visual and olfactory cues using combinations of the odors from the two flower species, *D. wrightii* and *A. palmeri*, and visual stimuli including conical paper flowers – that serve as an attractive visual stimulus while controlling for morphological and tactile differences between flower species –, and bagged flowers. Last, using experienced moths, we tested the importance of olfactory and visual cues when the floral stimuli were the same (coupled), or were switched (uncoupled) from that previously experienced by the moths (Table 1). Unless otherwise stated, we define a behavioral “response” as the first proboscis extension by the moth into one of the treatments in the two-choice assay.

### Behavior of naïve moths

#### Single modality trials

**Figure 5:**
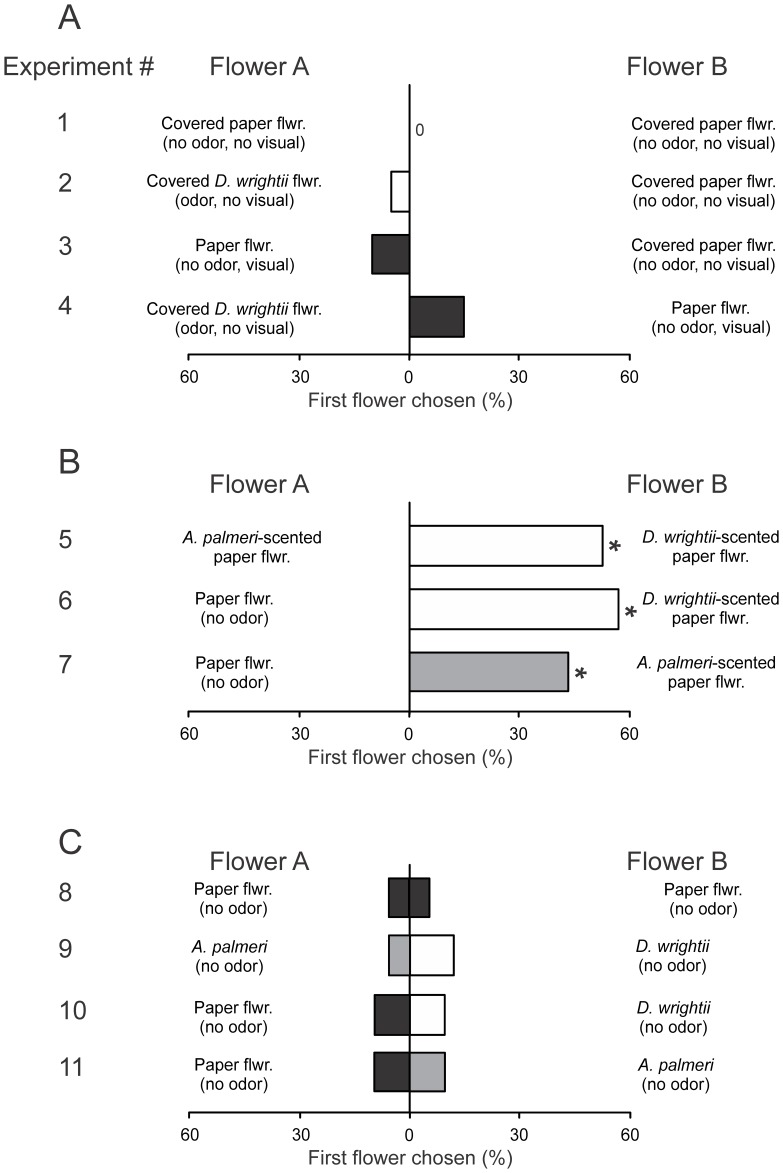
Two-choice experiments examining the visual and olfactory floral preferences of naïve male *M. sexta* moths. (**A**) Using a green shade-cloth to mask the visual display of the flowers, the effects of scent and a visual stimulus (paper flower) were tested in isolation and simultaneously. (**B**) With artificial flowers, the percentages of moths that chose paper flowers emitting *D. wrightii* (white bars), *A. palmeri* scent (grey bars), or no scent (control) flowers (black bars). (**C**) With bagged flowers to stop the scent emissions but allowing display of the floral visual signals, the percentages of moths that chose the *D. wrightii*, *A. palmeri*, or the (control) paper flower visual display. Asterisks (*) denote a significant deviation from a random distribution (*G*-test: *P*<0.05). 20–40 moths were used in each two-choice experiment. Moths were tested individually, with each two-choice treatment using different groups of moths. *D. wrightii* flower cues (odor, and/or visual) are represented by white bars, *A. palmeri* flower cues (odor, and/or visual) are represented by grey bars, and black bars represent the paper flower (no odor) control.

*M. sexta* moths have been shown to elicit feeding responses when presented with a visual stimulus [34], but moths are strongly attracted to stimuli that display both visual and olfactory cues [18]. We thus predicted that responses to a visual stimulus are greater when the moth is also exposed to an olfactory stimulus. In addition, we predicted that a visual stimulus is required for the proboscis extension response. In Experiment 1, moths did not behaviorally respond to treatments that lacked a visual and olfactory stimulus (*n* = 0/20; Fig. 5A). However, in Experiments 2 and 3, the presence of either a visual (paper flower) or olfactory stimulus (shade-cloth covered paper flower emitting a floral scent) elicited low-level responses in behavior (5 and 10%, respectively) in comparison to the shade-cloth covered paper flower (Fig. 5A). In Experiment 4, when these two floral cues were tested against one another, the visual stimulus elicited a similar low-level response similar to Experiments 2 and 3. Against our predictions, moths responded to both the visual and olfactory cues alone, albeit at low levels (5–10%), and the presence of the odor cue in the arena did not significantly change the response to the visual stimulus (Fig. 5A; *G*-test: *G* = 1.04, *P* = 0.59).

#### Multimodal trials

To examine more closely the interplay of olfactory and visual cues on behavior, and how the natural visual stimulus of the flower modifies behavior, experiments were performed using paper and bagged flowers as the visual stimuli, and the natural emission of the flowers as the olfactory cues. These experiments allowed us to test the prediction that the visual display of the natural flowers elicits a greater response than the conical paper flowers (that lacks the morphological features of the *D. wrightii* flower or *A. palmeri* umbel). Furthermore, these experiments allowed us to test the prediction that moths respond to the visual and olfactory displays of *D. wrightii* flower over the displays of the *A. palmeri* umbel.

Results from these experiments showed that floral odors elicited robust feeding responses in *M. sexta* moths. When moths were presented with a choice between a *D. wrightii*-scented paper flower and *A. palmeri*-scented paper flower (Experiment 5), moths first attempted to feed from the *D. wrightii* flower (Fig. 5B; *G-*test for first choice: *G* = 12.47, *P*<0.001) and also spent more time attempting to feed from that flower (paired *t*-test: *t* = 5.29, *P*<0.001). However, when moths were exposed to an unscented and scented paper flower (Experiments 6 and 7), irrespective of whether it was scented from *A. palmeri* or *D. wrightii*, they were significantly more likely to respond to the scented flower (Fig. 5B; *G-*test: *G*>8.61, *P*<0.01). By contrast, when moths were exposed to the bagged flowers – thereby eliminating the floral scent but maintaining the visual signals – (Experiments 8–11), only a few of the moths responded to the stimuli (ca. 10%) with no significant difference between flower species (Fig. 5C; *G-*test: *G* = 0.68, *P* = 0.41), although moths spent slightly more time probing the *D. wrightii* flower than the *A. palmeri* umbel (5.85 and 2.42 s for *D. wrightii* and *A. palmeri*, respectively).

**Figure 6:**
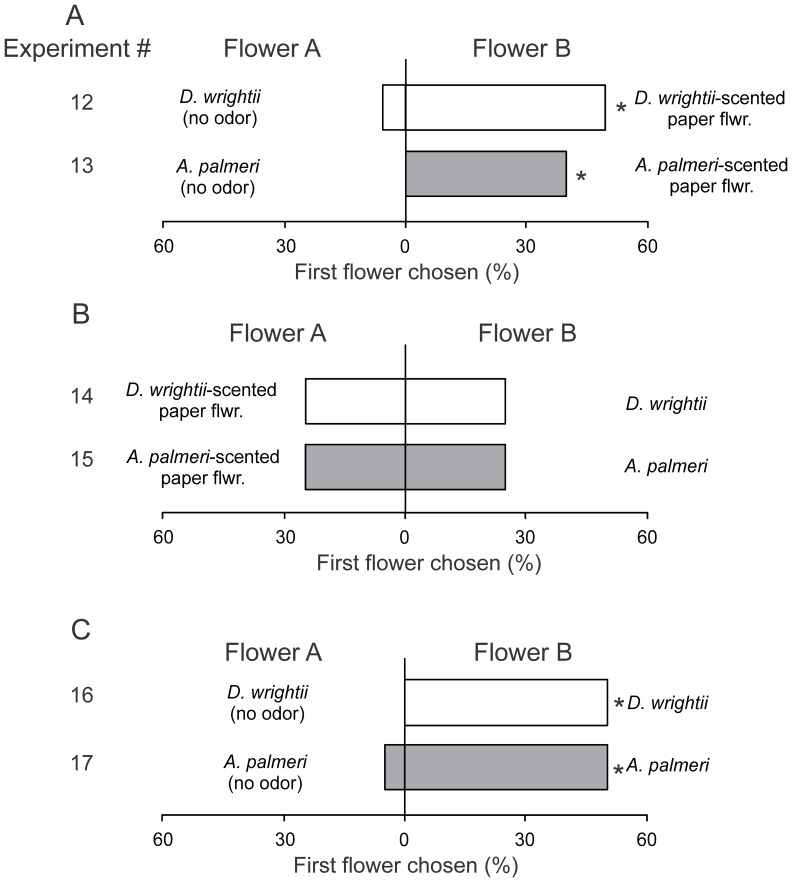
Two-choice experiments examining the single modality and multimodality floral display preferences in naïve male *M. sexta* moths. (A) With visual (bagged flowers) *versus* olfactory (scented paper flowers) displays, the preferences in naïve moths using *D. wrightii* and *A. palmeri* olfactory and visual displays. (B) With olfactory-dominant (scented paper flowers) and multimodal (olfactory and visual) flowers, the percentages of moths that chose the floral displays. (C) With visual-only (bagged flowers) and multimodal (olfactory and visual) flowers, the percentages of moths that chose the bagged (visual only) or multimodal (visual and olfactory) floral displays. *D. wrightii* flower cues (odor, and/or visual) are represented by white bars, and *A. palmeri* flower cues (odor, and/or visual) are represented by grey bars. Asterisks (*) denote a significant deviation from a random distribution (*G*-test: *P*<0.05). 16–30 moths were used in each two-choice experiment. Moths were tested individually, with each two-choice treatment using different groups of moths.

**Figure 7:**
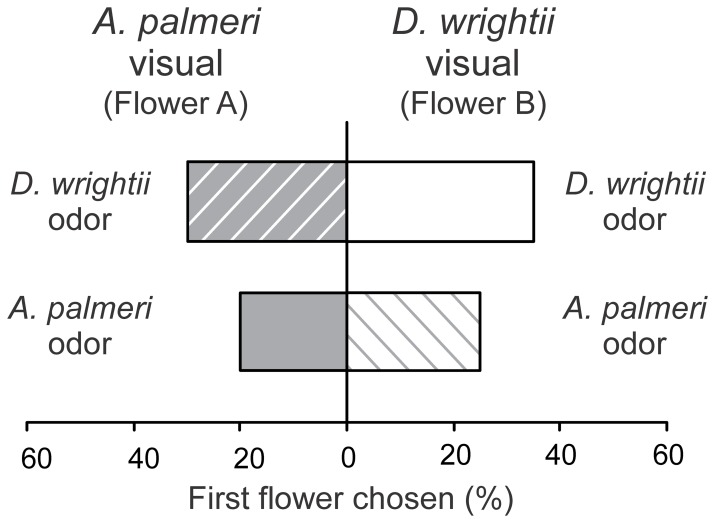
Two-choice experiments examining the visual floral preferences of naïve male *M. sexta* moths when the floral scents are similar. (*Top*) The percentages of moths that chose the the *A. palmeri* visual display (Flower A; grey bar, hashed white) or *D. wrightii* visual display (Flower B; white bar) when both flower species emit the *D. wrightii* scent. (*Bottom*) The percentages of moths that chose the *A. palmeri* visual display (Flower A; grey bar) or *D. wrightii* visual display (Flower B; white bar, hashed grey) when both flower species emit the *A. palmeri* scent. 20 moths were used in each two-choice experiment. In both experimental series, there were no significant differences in the first flower chosen (*G*-test: *P*>0.73).

When moth feeding behaviors were examined in response to choices between single or multiple modalities, it became clear that the flower odors were important for mediating naïve moth behaviors, but visual stimuli, regardless of whether they came from a cut flower or a conical paper flower, were also important. In Experiments 12 and 13, moths were significantly more likely to respond to scented paper flowers compared to unscented (bagged) flowers (Fig. 6A; *G*-test both choice treatments: *G*>8.57, *P*<0.01) and spent more time attempting to feed from the paper flowers (paired *t*-test both choice treatments: *t*>2.36, *P*<0.05). Similarly, in Experiments 16 and 17, moths significantly responded to, and spent more time attempting to feed from, flowers with the combination of olfactory and visual signals over unscented (bagged) flowers with just the visual stimuli (Fig. 6B; *G-*test both choice treatments: *G*>8.54, *P*<0.01; paired *t*-test both choice treatments: *t*>3.03, *P*<0.05). Moreover, behavioral responses to the combination of olfactory and visual cues did not significantly differ from the scented paper flowers (Fig. 6C; *G-*test: *G*<0.01, *P*>0.99: Experiments 14 and 15), and although moths spent slightly longer attempting to feed from flowers with the multimodal odor and visual display compared to the paper flowers emitting a floral scent (mean response time of 5.0 and 3.6 s, respectively), this difference was not significantly different (paired *t*-test: *t*>0.55, *P*>0.33). Lastly, in Experiments 18 and 19, when moths were presented with a uniform olfactory cue with the visual displays of both flower species there were no significant differences in the first flowers chosen or the time spent feeding (*G*-test for both choice tests: *G*<0.11, *P*>0.73; paired *t-*tests for feeding time in both choice tests: *t*<0.66, *P*>0.52), although there was a slight, but not statistically significant, response to the *D. wrightii* visual display (Fig. 7). In all experimental series, moths had similar average feeding times (Fig. 4), indicating that they actively sought to feed from the floral stimuli.

**Figure 8:**
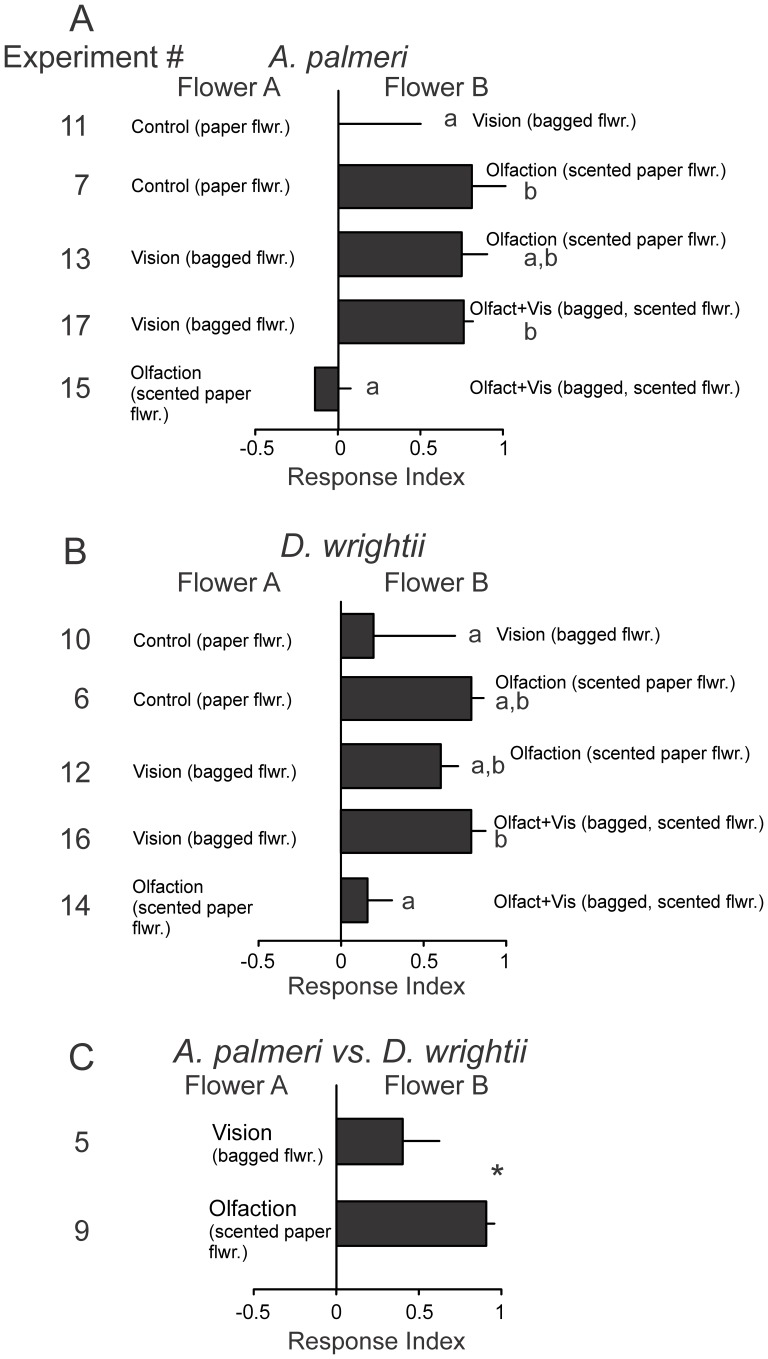
Response indices calculated from the time moths spent attempting to feed from the flowers in the single modality versus control, or single modality versus multimodal two-choice experiments. (A) *A. palmeri* floral signals. (B) *D. wrightii* floral signals. (C) *A. palmeri* versus *D. wrightii* visual (*top*) and olfactory (*bottom*) signals. Letters (A,B) or asterisks (C) denote a significant difference between two-choice treatments (unpaired *t*-test: *P*<0.05).

A Response Index was calculated based on the time moths spent attempting to feed from flowers (Time_FlowerB_ – Time_FlowerA_)/(Total Time_FlowerB+A_) to examine in detail the differences between flower species and the contribution of olfactory and visual cues. Based on this index, we first examined the relative effects of single and multiple modalities on moth feeding responses to the *A. palmeri* umbel. Results from these comparisons demonstrated a significant difference between olfactory and visual cue with the naïve moths displaying no attraction to the *A. palmeri* visual display (Fig. 8A). Similarly, moths displayed a slight, but not significant, attraction to the *D. wrightii* visual display in comparison to the paper flower, and an attraction to the multimodal (visual and olfactory) signals (Fig. 8B). Finally, examination of the moth Response Indices between visual and olfactory signals and flower species demonstrated a strong attraction to the *D. wrightii* olfactory signal relative to *A. palmeri*, and a slight attraction to the *D. wrightii* visual signal (Fig. 8C).

#### Experienced moth behavior and floral cue coupling

When foraging in the field moths learn to associate visual, morphological, and olfactory floral signals with the nectar rewards. We therefore examined how learning modified the behaviors of hawkmoths in response to specific combinations of floral signals, especially when the signals were modified from those that were previously experienced. These experiments allowed us to test the prediction that moths learn both visual and olfactory flower cues, and that both are equally important in mediating foraging decisions. To test this prediction, moths that had fed from real flowers the previous evening were re-tested the next evening to treatments where the visual and olfactory signals were maintained based on the flower species (coupled), or were switched between species (uncoupled).

**Figure 9:**
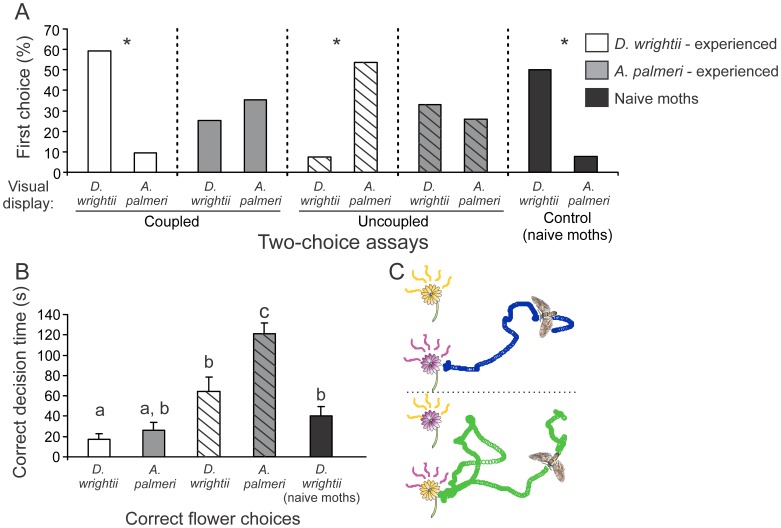
The effects of coupled (solid bars) versus uncoupled (hashed bars) floral displays for experienced male *M. sexta* moths. 24 hours prior to testing, moths were assigned to one of three treatment groups: moths exposed to *D. wrightii* flowers (white bars), *A. palmeri* flowers (grey bars), or flower-naïve moths (black bars), and were re-tested the following evening. (A) Using either real flowers with their visual and olfactory signals coupled, or uncoupled, the percentages of moths (from the total number of moths tested) that chose *D. wrightii* or *A. palmeri* flowers. An asterisk (*) denotes a significant deviation from a random distribution (*G*-test: *P*<0.05). (B) The time moths spent flying before attempting to feed from the previously experienced olfactory floral cue. Letters denote a significant difference between two-choice treatments (unpaired *t*-test: *P*<0.05). (C) Two-dimensional flight tracks for experienced moths to coupled floral displays (*top*, blue circles) and uncoupled floral displays (*bottom*, green circles) in a flight arena. Circles correspond to video images captured at 0.033 s intervals. Moths were tested individually, with each two-choice treatment using different groups of moths.

Moths that had previously experienced *D. wrightii*, when exposed to either a *D. wrightii* flower or *A. palmeri* umbel, significantly responded to the *D. wrightii* flower (Fig. 9A; *G-*test: *G*>6.19, *P<*0.05). Moths that had experienced the *A. palmeri* umbel, however, did not significantly respond to one flower over the other based on the first flower chosen (*G*-test: *G* = 0.33, *P* = 0.56). However, when the floral signals were switched – that is, when the *D. wrightii* scent was paired with the *A. palmeri* visual display, or *vice versa* – moths ultimately responded to the first flower based on an association with the olfactory, rather than the visual, signal (Fig. 9A). The time it took moths to make the correct decision based on the olfactory signal, however, was significantly higher for experienced moths in the uncoupled two-choice treatment than naïve and experienced moths in the coupled two-choice treatment (Fig. 9B; unpaired *t-*test: *t* = 5.71, *P*<0.001). Similarly, the total distance of the moth flight tracks to the uncoupled floral treatments were significantly longer than those in the coupled treatments (mean±se = 12.0 ± 1.7 and 5.7 ± 0.8 m for uncoupled and coupled treatments, respectively; unpaired *t-*test: *t* = 3.27, *P*<0.01). Experienced moths would quickly orient and fly to the coupled floral display, which contrasted with the behavior of experienced moths in the presence of the uncoupled display in which moths flew back and forth between the two treatments before ultimately making a proboscis extension (Fig. 9C). Thus, although moths learned to associate the nectar reward with the olfactory signal, the visual display was also learnt during this process.

## Discussion

Floral displays are excellent examples of adaptive evolution to potential pollinators, with the individual traits targeting specific pollinator sensory modalities such as vision and/or olfaction [48]. Single traits or suites of floral traits can act as key determinants of reproductive success for plants by attracting effective pollinators to visit and subsequently transport pollen. For the naïve *M. sexta* moths, olfactory signals play a critical role in mediating flower visitation and feeding that evoke either innate (*D. wrightii*) or learned (*A. palmeri*) behaviors, although the visual display of the highly attractive *D. wrightii* flowers did increase the Response Indices by approximately 20% when combined with the olfactory signal. Our prediction that the *D. wrightii* displays (visual and olfactory) were more attractive than the *A. palmeri* displays was verified by the behavioral responses to the olfactory display, but the *D. wrightii* visual display only elicited slightly longer feeding times (Fig. 5). Furthermore, although the semi-natural visual display of the flowers elicited longer feeding times than the paper flowers, this result was not statistically significant (Fig. 4). The influence of the flower's visual display was magnified, however, when moths had previously learned to associate the floral signals – both olfactory and visual – with the nectar reward. By ‘uncoupling’, or switching, the visual and olfactory displays of the two flowers, we were able to test the strength of the two learned modalities in mediating moth foraging decisions. In contrast to our prediction that the olfactory and visual displays were equally learned and used to drive behavior, moths made their first feeding response to the previously experienced flower odor, and not visual, cue. However, moths spent significantly longer time making this decision. Thus, suites of floral traits will have combinatorial effects on pollinator behaviors particularly when the traits are previously experienced.

### Learning and response time to multiple signals

Using multiple cues is thought to increase signal detection and discrimination by the receiver, and/or to increase learning and memory [49], [50]. Results from the current study demonstrated that prior experience decreased the time for moths to make the appropriate flower choice, mediated through the association of olfactory signals and nectar reward. Similar changes in feeding responses as a function of prior experience have been shown in *M. sexta*, where a single feeding act modified the moth's color preference, and the color preference persisting for several days [34]. In this study, we found that moths learned the flowers primarily based on the olfactory stimulus, but that the olfactory and visual stimuli together decreased the time it took for the moths to make a correct decision (Fig. 9). Beyond work with moths, increases in foraging efficiency as a function of prior experience has been found in other arthropods, such as bees and spiders, and vertebrates including mammals [3], [24], [51]–[53]. The ability to accurately discriminate between flowers may correspond to an increased caloric intake of nectar [54], and reduction of visits to non- or less-rewarding flower species [55].

### Pollinator attraction and learned responses to floral cues

Many pollinators exhibit attraction to certain suites of floral cues, and have the cognitive and neural machinery for the processing of these cues. When honeybees (*Apis mellifera*) are trained to achromatic stimuli they have preferences for flowers with visual wavelengths in the bee uv-blue and bee green range (approximately 410 and 530 nm, respectively). However, after repeated training to a chromatic stimulus, these preferences were extinguished [56]. The pipevine swallowtail butterfly, *Battus philenor*, has innate color preferences for yellow flowers [57], and hawkmoths (*Manduca* sp.) prefer highly reflective (450 to 600 nm), sweet-smelling flowers [40]. Pollinator preferences are reflective of specialization of the sensory system. For instance, *M. sexta* moths preference for flowers emitting oxygenated aromatics – which give the floral scent its sweet smell – can be attributed to the number of olfactory sensilla on the antenna, as well as the number and processing of projection neurons in the moth's antennal lobe, that are sensitive to those odorants [42], [58], [59].

### Floral odors and pollinator interactions

Nocturnal hawkmoths are important flower visitors for both *D. wrightii* and *A. palmeri*, and the contrast in floral traits between the two species may serve to increase the constancy of these associations, particularly for *A. palmeri* which is associatively learned by the moths [36], [37], [41]. Although *A. palmeri*'s floral traits have been interpreted as adaptations to bat pollination [37], [60], [61], these traits do not exclude other nocturnal taxa. In fact, *A. palmeri* produces hexose-rich nectar (>50 µl/h), emits a floral scent composed of monoterpenes and aliphatic compounds which are known to be attractive to diverse insects, and has brush-like flowers that permit nectar access by many animal taxa [20], [37], [60], [61]. As such, *A. palmeri* functions as an important nectar resource in southern Arizona for hawkmoths as well as the larger pollinator community, particularly prior to the onset of the summer monsoon rains when *D. wrightii* and other herbaceous plants are still dormant [37], [62].

The generalized associations between *A. palmeri* and its pollinators raises the question of which odorants may be under selective pressure by the hawkmoths, as well as other members of the pollinator assemblage. Furthermore, which of those odorants are easily learned by specific pollinators? Experiments with honeybees have shown that mixtures are learned by honeybees in a non-linear manner with respect to the individual constituents, but that certain odorants in the mixture are dominant over others. The odorant dominance may be context dependent whether or not certain other odorants are in the mixture [63], [64]. In a similar manner, *M. sexta* processes the *A. palmeri* odor as a function of only five odorants in its complex bouquet, and the five odorants are sufficient to reproduce the behavior elicited in response to the complete mixture comprised of 60 odorants. These results suggest that pollinators may be processing and learning floral mixtures as a few select compounds, and that individual pollinators – with their sensitivity to specific odorant classes – may be selecting for different odorants in the bouquet. These results may explain, in part, the complexity of the *A. palmeri* floral scent that attracts a diverse pollinator assemblage. Future work, in combination with the visual display, may disentangle the contribution and mechanisms of olfactory and visual channels in mediating pollinator learned and innate responses.
